# Allergic Fungal Otomastoiditis: A Case Report

**DOI:** 10.7759/cureus.45446

**Published:** 2023-09-18

**Authors:** Hiroshi Sakaida, Kazuhiko Takeuchi

**Affiliations:** 1 Department of Otorhinolaryngology-Head and Neck Surgery, Mie University Graduate School of Medicine, Mie, JPN

**Keywords:** fungus, corticosteroids, subtotal petrosectomy, allergic fungal rhinosinusitis, allergic fungal otomastoiditis

## Abstract

Otomastoiditis caused by an allergic reaction to fungi in the middle ear is rare, with only four cases reported in the English literature. We report the case of a patient with allergic fungal otomastoiditis. A 28-year-old man presented with otalgia, hearing loss, and vertigo. Exploratory tympanotomy revealed mucin with a peanut butter-like consistency and containing eosinophils and *Candida parapsilosis*, but no evidence of direct tissue invasion by fungi. The patient was treated with a combination of surgery and medication. Subtotal petrosectomy was finally performed to remove the middle ear mucosa and separate the middle ear from the external environment. Short-term prednisolone and long-term fluconazole were administered without satisfactory therapeutic results. The inflammatory condition has improved but continues without complete remission. Allergic fungal otomastoiditis is an extremely rare condition that may share pathophysiological features with allergic fungal rhinosinusitis, so a thorough examination combining bacterial cultures, histopathological examination with fungal staining, and serum antigen-specific immunoglobulin E against multiple fungi is essential. Optimal treatment probably comprises appropriate surgery and long-term administration of systemic corticosteroids. Definitive diagnostic criteria and therapeutic strategies need to be established, based on the accumulation of similar cases.

## Introduction

Otomastoiditis is an inflammatory condition of the tympanic cavity and mastoid air cells with a broad range of etiologies [1-5]. One common cause is bacterial infection. The diagnosis is typically straightforward via otoscopic findings, radiological examination, and bacterial cultures. Treatment generally involves the administration of antibacterial agents and surgery to drain infectious contents from the middle ear. Fungal infection can similarly cause otomastoiditis [3,4].

However, otomastoiditis caused by an allergic reaction to fungi in the middle ear is rare, with only four previous cases reported in the English literature [6-9]. This disease entity is termed allergic fungal otomastoiditis (AFOM), and the mechanism of pathogenesis seems similar to that of allergic fungal rhinosinusitis (AFRS) in the paranasal sinus. In addition, allergic bronchopulmonary aspergillosis in the lower airway likely involves similar pathogenetic mechanisms [10]. Shared pathophysiological features may exist among these entities in the ears, nose, and lungs, but anatomical and histological differences would affect the natural course of these three diseases and necessitate specific therapeutic strategies. In this paper, we report our experience with a patient with refractory otomastoiditis, probably due to an allergic reaction to fungi colonizing the middle ear.

## Case presentation

A 28-year-old man presented with a 3-week history of severe otalgia in the right ear. The patient also experienced vertigo and dizziness when applying positive pressure to the right external auditory meatus. We observed spontaneous nystagmus toward the left. The patient had a history of asthma during childhood but did not have asthma in adulthood. The patient had no signs or symptoms suggestive of upper or lower respiratory tract infections, such as nasal obstruction, rhinorrhea, postnasal drip cough, sore throat, dyspnea, and fever. The patient had mild diabetes mellitus that did not require insulin therapy.

**Figure 1:**
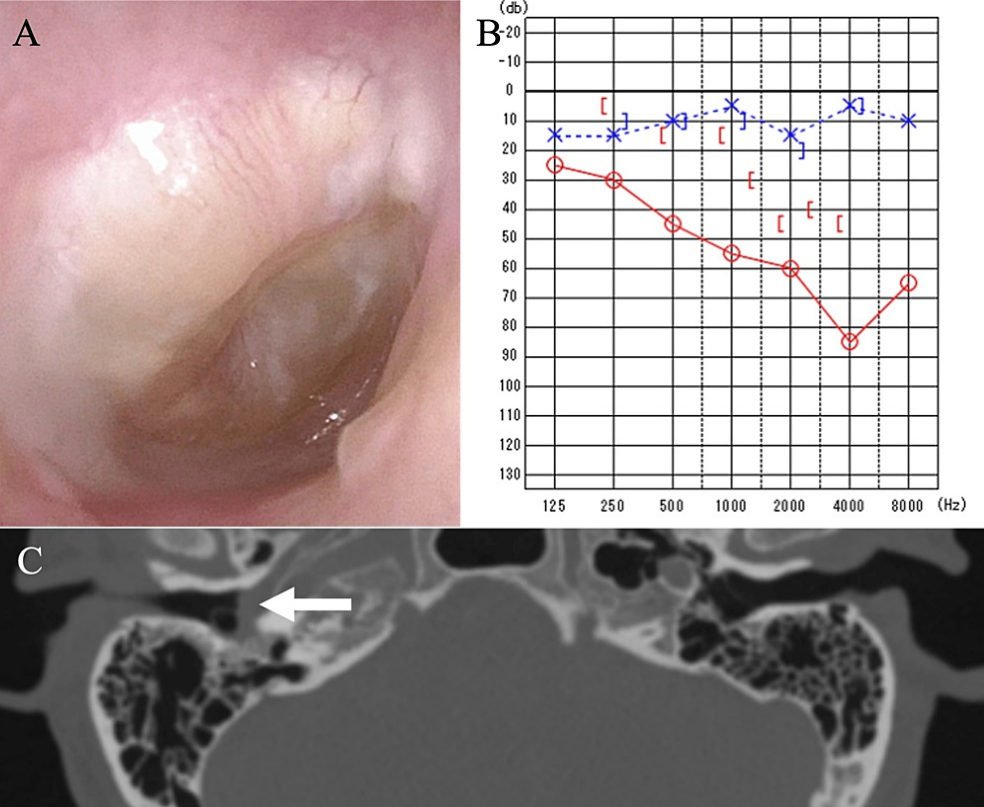
Otoscopic image, pure-tone audiogram, and computed tomography (A) Otoscopic image of the right ear at the first visit shows a bulging tympanic membrane with opaque fluid in the tympanic cavity. (B) Pure-tone audiogram at the first visit reveals a mixed hearing loss of 55 dB in the right ear (red line). (C) Axial computed tomography shows opacification of the right tympanic cavity without bony destruction (arrow).

The right tympanic membrane appeared bulging with opaque fluid in the tympanic cavity (Figure 1A). Pure-tone audiography showed mixed hearing loss of 55 dB in the right ear (Figure 1B). Computed tomography (CT) of the temporal bone revealed complete opacification in the tympanic cavity without destruction of the ossicles or any other bony structures (Figure 1C). The left ear appeared normal. Blood testing revealed an increased white blood cell count (10,180/μL; normal range 6,600-8,100/μL) and elevated C-reactive protein levels (1.3 mg/dL; normal <0.03 mg/dL).

We diagnosed otitis interna due to severe inflammation in the middle ear, but could not determine the exact pathogenesis. The patient underwent explorative tympanotomy to relieve the inflammation and identify the cause from fluid and mucosal samples from the tympanic cavity. The tympanic cavity was filled with thick, brown mucin that was hard to suction out because of its high viscosity. The mucosa of the tympanic cavity appeared thickened.

**Figure 2:**
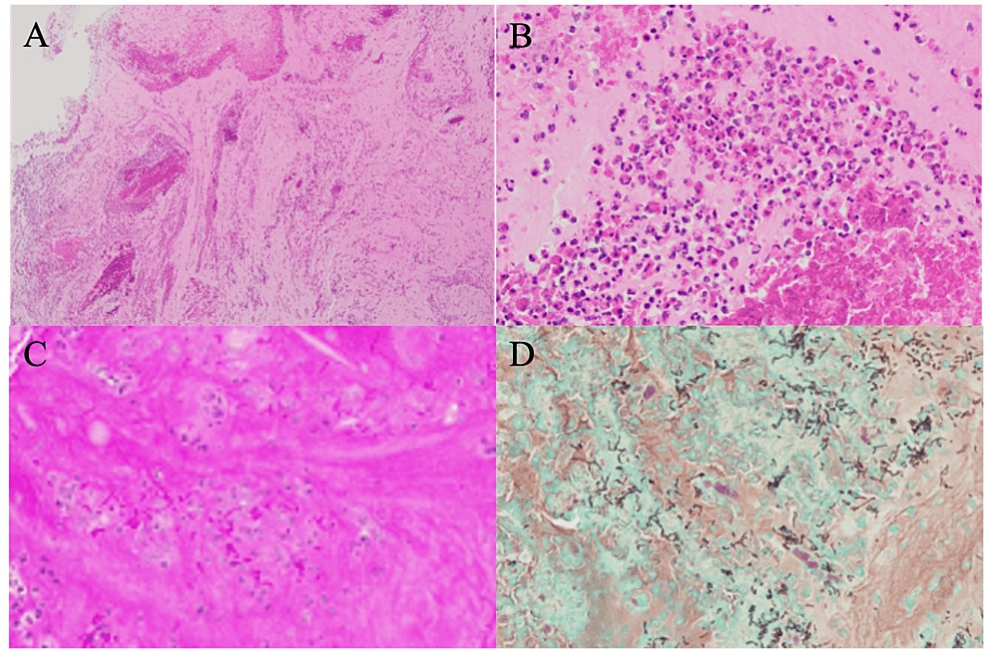
Histopathological slides of mucin from the right tympanic cavity The mucin contains diffuse infiltration of inflammatory cells (A: hematoxylin and eosin; magnification, 40×) and dense infiltration of eosinophils (B: hematoxylin and eosin; magnification, 400×). (C) Periodic acid Schiff staining reveals fungi, which stain purple (magnification, 400×). (D) Grocott methenamine silver staining reveals gray-stained yeast-like fungi (magnification, 400×).

Pathological examination conducted on the mucin revealed neutrophils, eosinophils (Figures 2A, 2B), and yeast-like fungi (Figures 2C, 2D). However, yeast-like fungi were not identified in the mucosa on histopathological examination with fungal stains such as periodic acid-Schiff staining and Grocott methenamine silver staining.

Bacterial culture of the mucin grew Candida parapsilosis. Serum antigen-specific immunoglobulin (Ig) E test results were positive for many fungal species, including *Alternaria, Cladosporium, Aspergillus, Candida, Penicillium, Trichophyton, Helminthosporium, *and *Mucorales*. Serum total IgE level was elevated (1,974 IU/mL; normal, <173 IU/mL). Although the white blood cell count was elevated, the percentage of eosinophils was 3.5% (normal range, 0.6-8.3%), again within normal limits. The β-D glucan level was likewise within the normal range (<0.3 pg/mL; normal, <11.0 pg/mL). No findings were suggestive of specific diseases such as tuberculosis or autoimmune disease.

**Figure 3:**
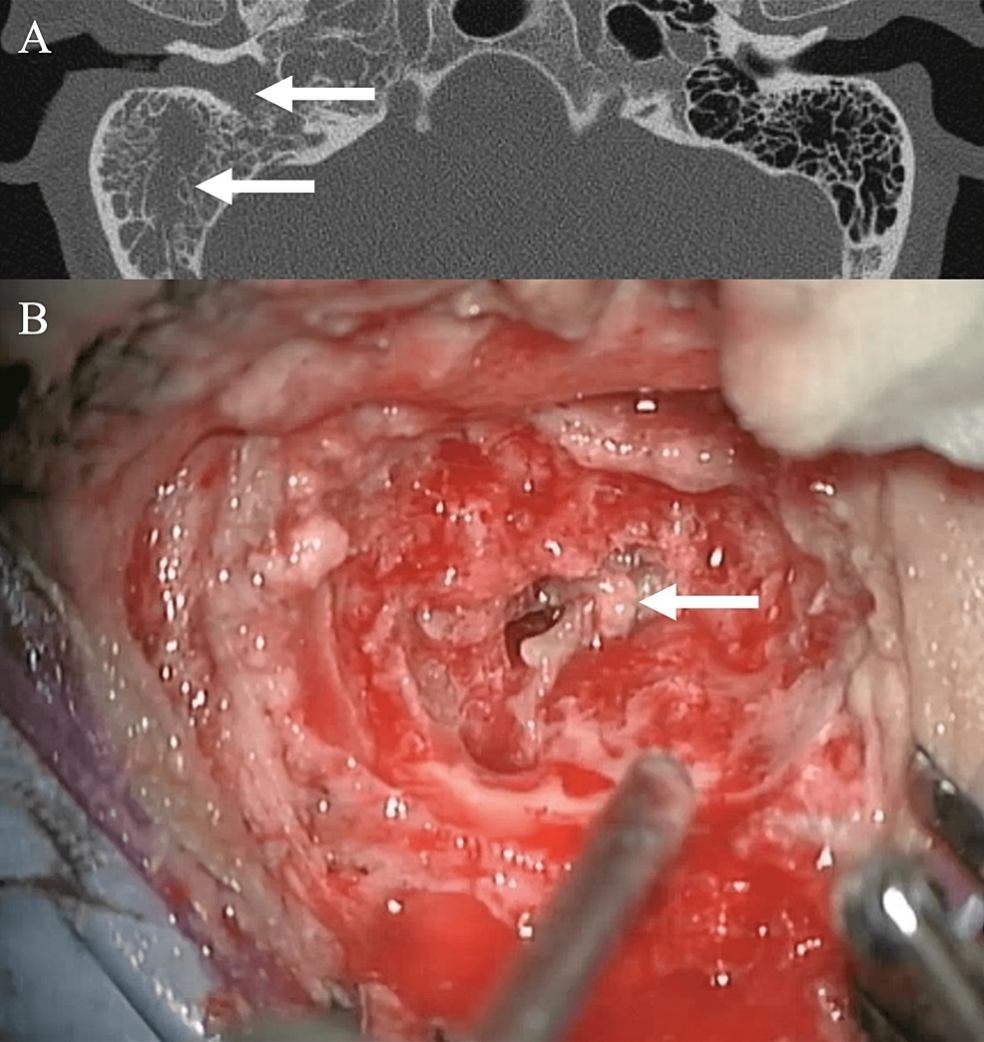
Computed tomography of the temporal bone and intraoperative photograph (A) Axial computed tomography shows opacification of the right tympanic cavity and mastoid air cells without bony destruction (arrows). (B) Intraoperative photograph shows contents with peanut butter-like thickness (arrow) filling the mastoid air cells and an edematous mucosa.

We tentatively concluded that an intense allergic inflammation directed against fungi colonizing the middle ear was probably responsible for the otitis media. We followed the standard treatment regimen for AFRS, starting with prednisolone at 20 mg/day. This was then stopped after 2 months when the underlying diabetes mellitus began to exacerbate with elevated levels of blood glucose. We also started fluconazole at 400 mg/day to suppress fungal proliferation. Even after administering these medications, the patient continued to complain of otalgia, dizziness, and hearing loss. Otoscopic examination showed fluid retention in the tympanic cavity. Repeat CT revealed complete opacification of the tympanic cavity and mastoid air cells, but no bony destruction (Figure 3A).

We again performed tympanomastoidectomy to remove pathological contents from the middle ear. Intraoperative findings revealed that the right tympanic cavity and the mastoid air cells were filled with thick, peanut butter-like mucin, and the mucosa was thickened (Figure 3B). We removed as much mucin as possible. The specimens were sent for bacterial and histopathological examinations, yielding the same results as those from the first surgery.

One year after the second surgery, the patient presented with otalgia, dizziness, hearing loss, and headache. We speculated that the remnant mucosa was the cause of inflammation, and that complete removal of the middle ear mucosa would be unavoidable to achieve symptom resolution.

The patient underwent a third surgery to eradicate the inflamed mucosa. We performed subtotal petrosectomy with the intention of eliminating the middle ear mucosa. Fluconazole was continued as adjuvant medical therapy, but systemic corticosteroids were not administered to avoid potential exacerbation of diabetes mellitus. Symptoms gradually improved without achieving complete remission and have continued for two years at the time of writing this report.

## Discussion

The pathophysiology of AFOM is considered analogous to that of AFRS since AFOM and AFRS share similar histopathological features. Fungal antigens may stimulate a type I and/or type III immunological response with eosinophilia producing allergic sticky mucin [11,12]. AFOM is a rare disorder with only four cases reported previously [6-9]. The first case was documented in 2012 by Chen and Chiang [7], who described findings of thick, dark, peanut butter-like mucus in the mastoid air cells and tympanic cavity. Eosinophils and fungi compatible with *Aspergillus* species were present in the mucin, suggesting an allergic reaction to fungus in the middle ear. The causative pathogens in the remaining three cases were documented as *Aspergillus* species with no evidence of direct invasion into the mucosal tissue [6,8,9]. The causative pathogen in our case was *C. parapsilosis*, which has pathogenicity in invasive infectious diseases. Why *C. parapsilosis *did not cause invasive infection rather than acting as an allergen is unclear. One explanation is that our patient had a propensity for an allergic reaction, supported by high titers of antigen-specific IgE against multiple fungal species.

Diagnostic criteria for AFOM have not yet been established because of the rarity of this pathology. Singh et al. presented a case of AFOM, reviewed previously reported cases, and proposed working criteria for diagnosing AFOM [6]. These working criteria consist of the following five items: 1. An immunocompetent individual with or without type 1 hypersensitivity history; 2. The presence of thick mucin with peanut butter-like consistency or polypoidal tissue in the tympanic cavity without other systemic involvement; 3. Heterogeneous signal intensity in the tympanic cavity with bony expansion and resorption on radiology; 4. Fungal elements in the background of allergic mucin without invasion on histopathology; and 5. Response to local or systemic steroid medication.

Our patient fulfilled four of these five items, with the exception being the third item. The accumulation of more AFOM cases is necessary to validate the proposed diagnostic criteria.

The differential diagnosis of AFOM is eosinophilic otitis media (EOM), which is characterized by viscous fluid containing abundant eosinophils in the tympanic cavity. The diagnostic criteria of EOM were proposed in 2011, which consist of one major and four minor items [13]. The major item is the presence of otitis media with effusion or chronic otitis media with eosinophil-dominant effusion. The four minor items consist of (1) Highly viscous middle ear effusion (MEE), (2) Resistance to conventional treatment for otitis media, (3) Association with bronchial asthma, and (4) Association with nasal polyposis. A definitive diagnosis requires the presence of two or more minor items in addition to the major item. According to the criteria of EOM, AFOM in the present patient can be classified under EOM. However, the diagnostic criteria of EOM do not refer to the presence of fungal elements in the allergic mucin. In this respect, AFOM might be classified as a specific form of EOM.

Regarding the involvement of fungi in the pathophysiology of EOM, antigen-specific IgE in MEE of EOM patients was studied by Kanazawa et al [14]. No fungal-specific IgEs were detected in the serum of EOM patients, but approximately 40% of EOM patients tested positive for one or more fungal antigens detected in MEE. Based on their results, antifungal IgE might be produced locally in the middle ear, suggesting a possible causality of fungi on EOM. However, they did not study the histopathology of the MEE and tissues in the tympanic cavity with a focus on fungi.

Given the possible similarity of pathophysiologies between AFOM and AFRS, the fundamental methods of management applied to patients with AFRS appear reasonable to use for patients with AFOM [11]. Therapeutic options include surgery and pharmacotherapy. However, the anatomical and histological differences may require specific therapeutic strategies. Surgical intervention is aimed at removing mucin containing the causative fungal antigen and establishing an effective ventilation route for the middle ear. In our patient, canal wall-up tympanomastoidectomy was performed to remove mucin, but yielded unsatisfactory results, and the disease persisted. We therefore reviewed our therapeutic strategy to eradicate the mucosa, which produces mucin and recruits inflammatory cells such as eosinophils. Simple tympanostomy tube placement might have been effective as an initial intervention in the early stage of the disease to provide a ventilation route to the outside of the tympanic cavity. 

We believe that subtotal petrosectomy was the most suitable surgical technique for our patient in the late phase of disease progression. Subtotal petrosectomy comprises three main procedures [15]. The first step involves the complete removal of the mastoid air cells and the mucosa lining the middle ear cavity. The second step is blind sac closure of the external auditory canal and obliteration of the Eustachian tube to separate the middle ear cavity from the external environment. The final step is the obliteration of the middle ear cavity with abdominal fat. In retrospect, whether subtotal petrosectomy could have thoroughly eliminated the targeted lesions in our patient is uncertain. The presence of fungal allergens and remnant mucosa after subtotal petrosectomy may have worsened the disease course.

The mainstay medication is corticosteroids. In AFRS, topical and systemic corticosteroids are essential parts of pharmacotherapy before and after surgery [12]. Suppressing inflammatory responses in the sinuses is complementary to the surgical removal of eosinophilic material and fungal debris. Long-term, low-dose systemic steroids are sometimes required to obtain a durable response. Systemic corticosteroids also appear reasonable for AFOM. However, we did not administer systemic corticosteroids for a long period because of the risk of exacerbating the underlying diabetes mellitus. The result was inadequate therapeutic efficiency. In retrospect, corticosteroids should be given along with strengthened control of diabetes mellitus. Antifungal agents have been investigated in many studies of AFRS [16]. However, a Cochrane systematic review examining the use of topical and oral antifungals in patients with AFRS was unable to make any recommendation because of the low quality of evidence [16]. Hence, insufficient evidence exists to recommend for or against antifungal therapy in AFRS.

## Conclusions

We presented a case of AFOM, representing the fifth report in the English literature. AFOM is rare, but should be considered in patients presenting with otomastoiditis with thick and dense mucin in the middle ear. Bacterial culture and histopathological examinations with fungal stains are essential when this disease entity is suspected. Any evidence of hypersensitivity to fungi may help narrow the differential diagnosis. We treated the patient with multiple surgeries, short-term prednisolone, and long-term fluconazole, all of which resulted in insufficient therapeutic effect. Optimal treatment probably comprises appropriate surgery to eliminate the middle ear mucosa and long-term administration of systemic corticosteroids. Definitive diagnostic criteria and therapeutic strategies should be established with the further accumulation of similar cases.
